# Anti-hepatotoxic activities of *Hibiscus sabdariffa* L. in animal model of streptozotocin diabetes-induced liver damage

**DOI:** 10.1186/1472-6882-14-277

**Published:** 2014-07-30

**Authors:** David O Adeyemi, Victor O Ukwenya, Efere M Obuotor, Stephen O Adewole

**Affiliations:** Department of Anatomy and Cell Biology, Obafemi Awolowo University, Ile-Ife, Nigeria; Department of Anatomy, Ekiti State University, Ado Ekiti, Ekiti State Nigeria; Department of Biochemistry, Obafemi Awolowo University, Ile-Ife, Nigeria

**Keywords:** Liver, Toxicity, Diabetes mellitus, *Hibiscus sabdariffa*, Flavonoids, Antioxidant

## Abstract

**Background:**

Flavonoid-rich aqueous fraction of methanolic extract of *Hibiscus sabdariffa* calyx was evaluated for its anti-hepatotoxic activities in streptozotocin-induced diabetic Wistar rats.

**Methods:**

Diabetes Mellitus was induced in Wistar rats by a single i.p injection of 80 mg/kg b.w. streptozotocin (STZ) dissolved in 0.1 M citrate buffer (pH 6.3).

**Results:**

The ameliorative effects of the extract on STZ-diabetes induced liver damage was evident from the histopathological analysis and the biochemical parameters evaluated in the serum and liver homogenates. Reduced levels of glutathione (GSH), catalase (CAT), superoxide dismutase (SOD), and glutathione peroxidase (GPx) (3.76 ± 0.38 μM, 0.42 ± 0.04 U/L, 41.08 ± 3.04 U/ml, 0.82 ± 0.04 U/L respectively) in the liver of diabetic rats were restored to a near normal level in the *Hibiscus sabdariffa* -treated rats (6.87 ± 0.51 μM, 0.72 ± 0.06 U/L, 87.92 ± 5.26 U/ml, 1.37 ± 0.06 U/L respectively). Elevated levels of aspartate amino transferase (AST), alanine amino transferase (ALT) and alkaline phosphatase (ALP) in the serum of diabetic rats were also restored in *Hibiscus sabdariffa* -treated rats. Examination of stained liver sections revealed hepatic fibrosis and excessive glycogen deposition in the diabetic rats. These pathological changes were ameliorated in the extract-treated rats.

**Conclusion:**

The anti-hepatotoxic activity of *Hibiscus sabdariffa* extract in STZ diabetic rats could be partly related to its antioxidant activity and the presence of flavonnoids.

## Background

The liver plays a central and crucial role in the regulation of carbohydrate metabolism. Its normal functioning is essential for the maintenance of blood glucose levels and of a continued supply to organs that require a glucose energy source [1]. In addition, it has great capacity to detoxify toxic substances and synthesize useful ones. Therefore, the damage which is caused by hepatotoxic agents is of grave consequence to the body as it deprives the liver of its principal functions [2]. A significant amount of liver damage is induced by lipid peroxidation and other oxidative damages which are caused by the hepatotoxic chemicals [3, 4]. It has been reported that liver injury caused by a variety of deleterious agents induces inflammation, necrosis, fibrosis cirrhosis and functional deteriorations [5].

Diabetes is a chronic disease with a relatively high prevalence in many populations across the world [6]. Diabetes is associated with several structural and functional liver abnormalities that affect glycogen and lipid metabolism [7–9]. Excess glycogen deposition, fibrosis, cirrhosis, steatohepatitis and Biliary disease in the liver has been reported in 55 – 80% of diabetic patients [10].

Plants used in traditional medicine for the treatment of liver disorders are of great interest, as they may serve as potential sources for new therapeutic agents that could be applied in the management and prevention of hepatic injuries. For example, plants rich in different phytochemical derivatives such as triterpenes, flavonoids or polyphenols, have been reported to exhibit antihepatotoxic effects in experimental liver-injury models induced by different types of hepatotoxicants, such as carbon tetrachloride, cadmium, galactosamine, acetaminophen and thioacetamide [11, 12].

*Hibiscus sabdariffa* (HS) (family: Malvaceae) has been reported as an ethnomedicinal remedy for a variety of ailments including hypertension, hyperlipidaemia, obesity, diabetes, Jaundice liver and urinary problems [13]. It’s antihypertensive [14, 15] hypolipidaemic [16, 17] anti-obesity [18, 19] and anti-hyperglycemic [13, 17] effects have been confirmed in man and experimental animals and the possible mechanisms have also been delineated. Water-soluble extracts from *Hibiscus sabdariffa L.* contain several antioxidants, such as protocatechuic acid [20] and anthocyanins [21, 22]. Recent studies have shown the potentials of these antioxidants in the protection of liver in chemically-induced peroxidative liver damage [22–24]. Hence this study investigates the potential anti-hepatotoxic properties of the flavonoid-rich extract of *Hibiscus sabdariffa* in animal model of streptozotocin diabetes- induced liver damage.

## Methods

### Chemicals

All chemicals used were of analytical grade. STZ was purchased from Sigma-Aldrich (St. Louis, MO, USA) Biochemical kits for AST, ALT and ALP assay were purchased from Randox laboratory (Crumlin, Co. Antrim, UK); Biochemical kits for catalase, GSH, GPx and TBARS assay were purchased from Bio Assay System (Hayward, CA 94545 USA) while kit for SOD assay was purchased from Cell technology Inc (Mountain View, CA 94043). Other histological reagents and stains were purchased from Sigma-Aldrich (St. Louis, MO, USA).

### Animals

Healthy Wistar rats (200 – 250 g) of either sex obtained from the animal holding facility of the College of Healthy Sciences, Obafemi Awolowo University Ile-Ife were used for the experiment. They were maintained under standard environmental conditions of temperature, humidity and light and fed on standard rat pellets (Ladokun feeds, Ibadan, Nigeria) and water *ad libitum*. The animals were acclimatized to the laboratory for four weeks prior to the start of the experiment. The rats received humane care according to the USA’s National Institute of Health’s Guide for the Care and Use of Laboratory Animals [25] and their experimental use was approved by the animal Ethics Committee of Obafemi Awolowo University Ile Ife.

### Experimental design

The animals were randomly assigned into five groups A, B, C, D and E of twelve rats each. Group A was normal control (normoglycemic rats), group B was test group I (normoglycemic rats treated with *Hibiscus sabdariffa* calyx extract (HSCE)); group C was diabetic negative control (untreated diabetic rats given STZ as described in “Induction of Experimental Diabetes” section); group D was test group II (diabetic rats treated with HSCE); while group E was diabetic positive control (diabetic rats treated with protamin zinc insulin).

### Plant material

Matured calyxes of *H. sabdariffa* obtained from a local market in Ile-Ife, Nigeria were authenticated at the Forestry Research Institute of Nigeria (FRIN), Ibadan and a voucher specimen (FHI 107622) was submitted to the FRIN Herbarium for future reference.

### Extraction

Dried and pulverized calyxes of *H. sabdariffa* (200 g) were extracted three times with 70% methanol (500 mls × 3) with continuous stirring at room temperature for 24 hours each. The extract was concentrated *in vacuo* at 25°C using a vacuum rotary evaporator (RE 100B, Bibby Sterilin, United Kingdom) and the aqueous phase was partitioned with ethyl acetate (EtOAc). The aqueous fraction (coded HSCE) was freeze dried using a vacuum freeze drier (FT33- Armfield, England) and used for the experiment. Solvent elimination under reduced pressure and subsequent freeze-drying gave 11.75 g (i. e., 5.875% yield) of a dark red, powdery extract.

### Phytochemical analysis

The following phytochemical analysis was carried out on leaves of *Hibiscus sabdariffa* using the procedure of [26] as outlined below:

### Test for flavonoids

0.5 g of plant sample was suspended in 5 ml of water and 2.5 ml of methanol was added to it and filtered. 1 ml of NaOH 10% was added to 1 ml of the filtrate. The presence of a yellow precipitate indicated the presence of flavonoids.

### Test for tannins

7.5 ml of water was added to plant extract (1 g) and heated in a water bath. It was then filtered upon cooling. Few drops of iron III chloride (FeCl_3_) 0.5% were added to 2 ml of the filtrate. The appearance of a green or dark-blue precipitate indicated the presence of tannins.

### Test for alkaloids

2 g of plant sample was heated in a test tube containing 25 ml of HCl (1%) for 15 min in a boiling water bath. The suspension was then filtered and 5 drops of Meyer's reagent (potassium tetra iodomecurate) were added into the filtrate (1 ml). The formation of a precipitate indicated the presence of alkaloids.

### Test for saponins

0.5 g of plant extract was introduced into a test tube containing 7.5 ml of distilled water and the mixture heated for 5 min in a boiling water bath. The solution was then filtered and cooled to room temperature. Five millilitres of the filtrate was introduced into a test tube and agitated for 10 s. The formation of persistent foam indicated the presence of saponins.

### Test for triterpenes and steroids

0.5 g of plant sample was dissolved in chloroform (3 ml) and a few drops of acetic anhydride and concentrated H_2_SO_4_ were added. A purple coloration indicated the presence of triterpenes while bluish-green coloration indicated the presence of steroids. The formation of two layers upon addition of H_2_SO_4_ is characteristic of the presence of both triterpenes and steroids.

### Test for coumarins

One milligram of moistened sample was placed in a test tube and the test tube was covered with a filter paper moistened with 10% NaOH solution. After exposition of the paper to UV light for a few minutes, yellow green fluorescence was indicative of the presence of coumarins

### Acute toxicity study

Rats fasted for 16 h were randomly divided into seven groups of five rats each. One group served as control and received 0.3 mls of distilled water orally. The other groups (test groups) were treated with graded doses of HSCE; 200, 400, 800, 1600, 3200 and 6400 mg kg^-1^ dissolved in distilled water orally. Signs of toxicity (convulsion, hypoactivity, weakness, ataxia and salivation) and mortality in each cage were assessed 24 hours, 48 hours and 72 hours after administration of extract. LD_50_ of HSCE was determined in the rats using method of Abdel-Barry *et al.* [27].

### Induction of experimental diabetes

Animals were fasted (but still allowed access to water) for 16 hours prior to induction. Diabetes mellitus was induced in groups C,D and E rats by a single i.p. injection of 80 mg/kg bw STZ dissolved in 0.1 M sodium citrate buffer (pH 6.3) as previously reported [28, 29]. Group A rats were injected with equivalent volumes of citrate buffer i.p.

### Administration of drugs

Four weeks post induction of diabetes, daily doses of 1750 mg/kg bw HSCE was administered orally to the rats in test groups I and II (groups B and D) for 15 days by gavage while 1 IU/kg/day of protamine zinc insulin was administered i.p. to group E rats (diabetic positive control). Rats in group C (diabetic negative control) were left untreated.

### Terminal sacrifice procedures

A mid-line incision was made through the anterior abdominal walls of the rats under terminal chloroform anaesthesia. The liver tissues were excised and weighed. Some of the liver tissues were fixed in in 10% formol saline for 48 hours for histological procedures, some fixed in Bouin’s fixative for 24 hours for histochemical procedure while other parts were frozen for biochemical assay.

### Determination of liver weight

At sacrifice, the absolute liver weight was determined using a top loader sensitive balance (Mettler Toledo Germany). The relative weight of the liver (%) was calculated from the body weight at sacrifice and the absolute liver weights as previously described [29].


### Histological procedure

Liver tissues fixed in 10% formol saline were processed via paraffin wax embedding method [30]. Sections of 5 μm thickness produced were stained with haematoxylin and eosin (HE) for general histological examination of the liver tissues, Gordon and Sweets reticulin stain (GSR) [31] to histologically demonstrate reticulin fibres in the liver and with Masson trichrome stain (MT) to histologically demonstrate collagen fibres in liver. The sections were examined under Leica DM750 research microscope with a digital camera (Leica ICC50) attached. Digital photomicrographs were taken at various magnifications.

### Histochemical procedure

The tissues fixed in Bouin’s fixative were processed via paraffin wax embedding. Sections of 5 μm thickness produced were stained with periodic acid Schiff (PAS) with diastase control to histochemically demonstrate glycogen in the liver sections. Digital photomicrographs were also taken after examination under Leica DM750 research microscope

### Biochemical assays

#### Assay for liver function markers

Blood samples were obtained from the rats by cardiac puncture at sacrifice and were kept for 30 min at room temperature. Serum was separated from the blood samples by centrifugation at 5000 rpm for 10 min at room temperature. Serum marker of liver function such as such as aspartate amino transferase (AST) alanine amino transferase (ALT) and alkaline phosphatase (ALP) as well as serum total proteins (TP) and albumin (ALB) were estimated spectrophotometrically, using enzymatic colorimetric assay kits (Randox, Northern Ireland) following standard methods.

### Preparation of tissue homogenates

The excised livers were cut into separate portions for estimation of catalase (CAT), superoxide dismutase (SOD), glutathione peroxidase (GPX), glutathione (GSH) and thiobarbituric reactive subatances (TBARS). Tissues were rinsed in ice rinsed in ice-cold phosphate buffered saline pH 7.4. The tissues were then homogenized at 4°C in five volumes of homogenizing buffer per gram of tissue using a motor driven glass-Teflon Potter-Elvejhem homogenizer. The homogenizing buffer for CAT and GPX assay contained 50 mM tris HCl pH 7.5, 5 mM EDTA and 1 mM dithiotreitol (DTT); SOD assay homogenizing buffer contained 50 mM sucrose, 200 mM mannitol and 1 mM EDTA prepared in 10 mM Tris buffer pH 7.4.; GSH assay homogenizing buffer contained 50 mM potassium phosphate (PH 7.0) and 1 mM EDTA while the homogenizing buffer for TBARS Assay was a radio-immuno precipitation assay (RIPA) buffer which contained 150 mM sodium chloride, 50 mM tris buffer pH 8.0, 1.0% triton X-100, 0.1% sodium duodecyl sulphate (SDS) and 0.5% sodium deoxycholate. The homogenates were centrifuged in Harrier 15/80 MSE ultracentrifuge at 13,000 rpm for 15 minutes to obtain the supernatant.

### Biochemical assay for antioxidants and lipid peroxidation markers

The activities of CAT, SOD and GPX as well as the concentration of GSH and TBARS were determined in the resulting supernatants by a 96-well microplate-based assay using their specific quantitative colorimetric detection kits following manufacturers manual. Assay for catalase activity was based on catalase degradation of hydrogen peroxide using a redox dye [32]; the activity of glutathione peroxidase was measured in the tissue homogenates by measuring NADPH consumption in the enzyme-coupled reaction [33]; the activity of SOD was measured using sensitive colorimetric SOD assay kit which utilizes water soluble tetrazolium salt (WST-1) that produces a water-soluble formazan dye upon reduction with superoxide anion [34]; concentration of glutathione was measured based on the reaction of 5, 5’- dithiobis 2-nitrobenzoic acid (DTNB) with reduced glutathione to form a yellow product [35] while TBARS assay was based on the reaction of TBARS with thiobarbituric acid (TBA) to form a pink coloured product [36].

### Statistical analysis

All values were presented as mean ± standard error of mean (SEM) for twelve rats in each of the five group of rats. The significance of difference in the means of all parameters was determined using one-way analysis of variance (ANOVA; 95% confidence interval). Dunnett multiple comparison (DMC) and Student Newman-Keul’s (SNK) *post hoc* tests were carried out for comparison of all groups with control and comparison of all pairs of groups respectively. All statistics were carried out in GraphPad Prism. Values of p < 0.05 were considered as significant [37].

## Results

### Acute toxicity

No sign of toxicity was observed in all groups of animals tested within 72 hours. The LD_50_ of *H. Sabdariffa* in Wistar rats was found to be 3200 mg kg^-1.^

### Effects of HSCE on liver weight

The effects of HSCE on the body weight, absolute and relative liver weight in all groups of rats are shown in Table 1. The diabetic positive and negative controls presented with severe loss in body weight and absolute liver weight when compared with the other groups. However the relative weight of the liver with respect of the body weight was essentially similar in all groups.

**Table 1:** **Effects of** ***H. Sabdariffa*** **on body weight and liver weight**

	Body weight (g)	Absolute liver weight (g)	Relative liver weight (%)
A (normal control)	181.92 ± 6.49^†^	7.83 ± 0.64^†^	4.31 ± 0.33^†^
B (Test I)	179.75 ± 7.11^†^	7.93 ± 0.68^†^	4.41 ± 0.35^†^
C (Diabetic -ve control)	141.58 ± 3.33^*‡^	5.17 ± 0.51^*‡^	3.66 ± 0.29^†^
D (Test II)	168.17 ± 6.15^†^	7.02 ± 0.57^*§^	4.17 ± 0.32^†^
E (Diabetic + ve control)	139.41 ± 2.55^*‡^	5.08 ± 0.43^*‡^	3.64 ± 0.31^†^

*p < 0.05 compared with the normal control, determined by one way ANOVA followed by DMC *post hoc* test.

^†‡§^within column signifies p < 0.05 between groups with different symbols, determined by SNK *post hoc* test.

### Effects of HSCE on liver function markers

The activities of alanine amino transferase (ALT), aspartate amino transferase (AST) and alkaline phosphatase in normoglycemic, diabetic negative and positive controls and extract treated groups are presented in Table 2. The activities of AST, ALT and ALP significantly increased (p < 0.05) in the diabetic negative and positive control rats when compared with the normoglycemic rats. The activities of these liver marker enzymes were significantly lowered in Test group II rats. However, *H. Sabdariffa* had no effect on the activity of these enzymes in normal rats.

**Table 2:** **Effects of** ***H. Sabdariffa*** **on the liver function markers**

	AST (U/L)	ALT (U/L)	ALP (U/L)
A (normal control)	11.38 ± 2.46^†^	10.72 ± 1.64^†^	85.13 ± 7.08^†^
B (Test I)	12.05 ± 2.36^†^	10.63 ± 0.33^†^	78.62 ± 5.28^†^
C (Diabetic -ve control)	45.72 ± 5.28^*‡^	54.05 ± 5.03^*‡^	181.94 ± 12.12^*‡^
D (Test II)	14.68 ± 1.64^†^	12.08 ± 2.42^†^	103.21 ± 6.98^*§^
E (Diabetic + ve control)	37.33 ± 5.15^*§^	43.12 ± 4.16^*§^	149.06 ± 9.12^*‡^

*p < 0.05 compared with the normal control, determined by one way ANOVA followed by DMC *post hoc* test.

^†‡§^within column signifies p < 0.05 between groups with different symbols, determined by SNK *post hoc* test.

### Effects of HSCE on serum proteins

Elevated concentrations of TP and GLB in diabetic negative control rats were brought down significantly (p < 0.05) following administration of HSCE and insulin (Table 3). In addition albumin – globulin ratio which was significantly lower (p < 0.05) in the diabetic negative control group compared with normoglycemic rats was significantly raised in the diabetic positive control and test group II.

**Table 3:** **Effects of** ***H. Sabdariffa*** **on the serum proteins**

	Total protein (g/dl)	Albumin (g/dl)	Globulin (g/dl)	Albumin/globulin ratio
A (normal control)	8.19 ± 0.36^†^	4.26 ± 0.42^†^	3.93 ± 0.31^†^	1.08 ± 0.18^†^
B (Test I)	8.25 ± 0.26^†^	4.05 ± 0.18^†^	4.20 ± 0.26^†^	0.96 ± 0.21^†^
C (Diabetic -ve control)	13.28 ± 0.68^*‡^	4.12 ± 0.79^†^	9.16 ± 0.56^*‡^	0.49 ± 0.06^*‡^
D (Test II)	7.95 ± 0.19^†^	3.86 ± 0.24^†^	4.09 ± 0.41^†^	0.95 ± 0.11^†^
E (Diabetic + ve control)	8.21 ± 0.15^†^	3.93 ± 0.65^†^	4.28 ± 0.12^†^	0.92 ± 0.13^†^

*p < 0.05 compared with the normal control, determined by one way ANOVA followed by DMC *post hoc* test.

^†‡^within column signifies p < 0.05 between groups with different symbols, determined by SNK *post hoc* test.

### Effects of HSCE on liver antioxidants

The activities of the antioxidant enzymes (catalase, superoxide dismutase and glutathione peroxidase) and the concentration of glutathione (a non-enzymatic antioxidant) were significantly lower (p < 0.05) in the liver of the diabetic negative control rats compared with normoglycemic rats (Table 4). HSCE treatment elevated the activities of these antioxidant enzymes and concentration of glutathione significantly (p < 0.05) in the liver of test group II rats better than that demonstrated by protamin zinc insulin in the diabetic positive control group (Table 4). However HSCE treatment had no significant effects on these antioxidants in the liver of normoglycemic rats.

**Table 4:** **Effects of** ***H. Sabdariffa*** **on the liver antioxidants and lipid peroxidation markers**

	CAT (U/L)	GPx (U/L)	SOD (U/mL)	GLU (μM)	TBARS (μM MDA)
A (normal control)	1.04 ± 0.07^†^	1.71 ± 0.06^†^	101.42 ± 6.11^†^	8.97 ± 0.71^†^	7.79 ± 0.72^†^
B (Test I)	1.09 ± 0.06^†^	1.67 ± 0.08^†^	95.04 ± 6.56^†^	7.78 ± 0.67^†^	7.12 ± 0.46^†^
C (Diabetic -ve control)	0.42 ± 0.04^*‡^	0.86 ± 0.06^*‡^	41.08 ± 3.04^*‡^	3.76 ± 0.38^*‡^	17.56 ± 1.02^*§^
D (Test II)	0.72 ± 0.06^*§^	1.37 ± 0.06^*§^	87.92 ± 5.26§	6.87 ± 0.51^†^	8.63 ± 0.65^†^
E (Diabetic + ve control)	0.37 ± 0.03^*‡^	0.97 ± 0.05^*‡^	46.58 ± 3.57^*‡^	3.26 ± 0.39^*‡^	11.56 ± 0.73^*‡^

*p < 0.05 compared with the normal control, determined by one way ANOVA followed by DMC *post hoc* test.

^†‡§^within column signifies p < 0.05 between groups with different symbols, determined by SNK *post hoc* test.

### Effects of HSCE on lipid peroxidation

The concentration of TBARS, a marker of lipid peroxidation was significantly higher (p < 0.05) in the liver of diabetic negative control rats than in normoglycemic rats (Table 4). However HSCE treatment lowered the level of TBARS significantly (p < 0.05) in the treated rats.

### Histopathological assessment of the liver

**Figure 1:**
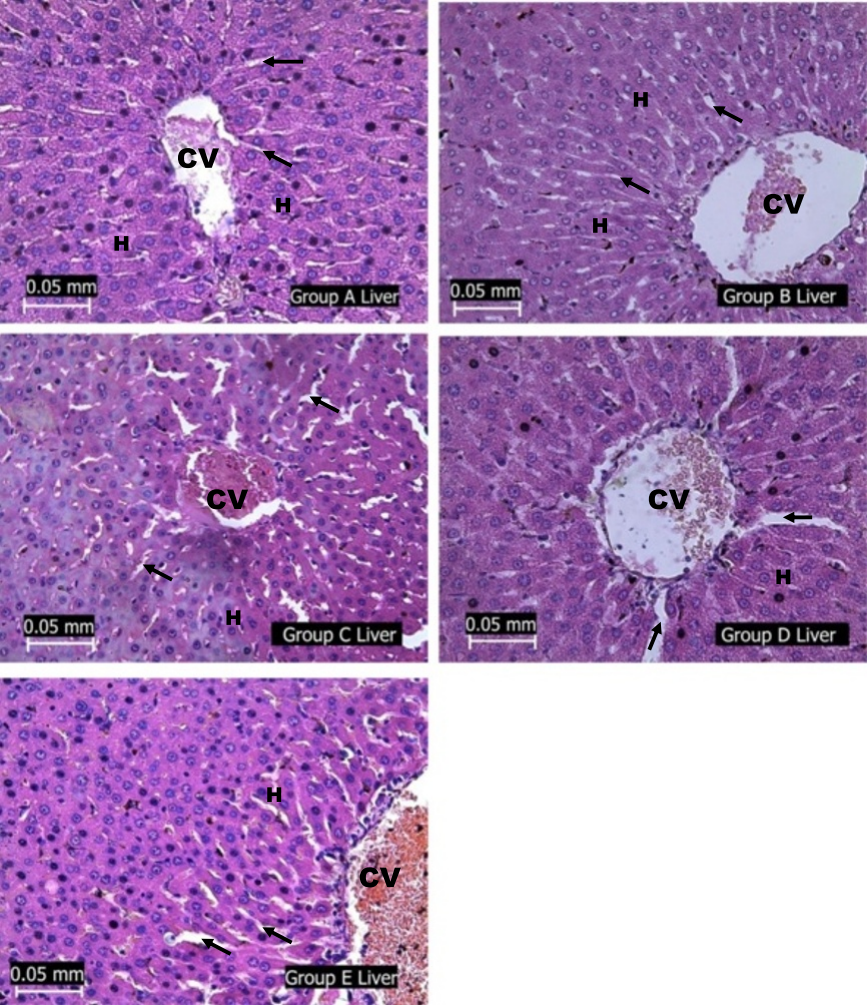
**Photomicrographs showing the liver of the experimental rats (A - normoglycaemic rats, B – test group I, C – diabetic negative control, D – test group II and E – diabetic positive control) stained with H & E.** Note the hepatocytes (H) arranged in plates around the central vein (CV) with sinusoids (arrow) in between the plates. Architecture of groups A B and D liver appeared normal. Distortion of architecture and signs of inflammation were observed in group C liver while group E liver section revealed a disheveled pattern of liver architecture with poorly defined hepatocytes and sinusoids except those in the immediate vicinity of the central vein. Vascular congestion of the central vein was also observed in the liver of group C and E rats.

**Figure 2:**
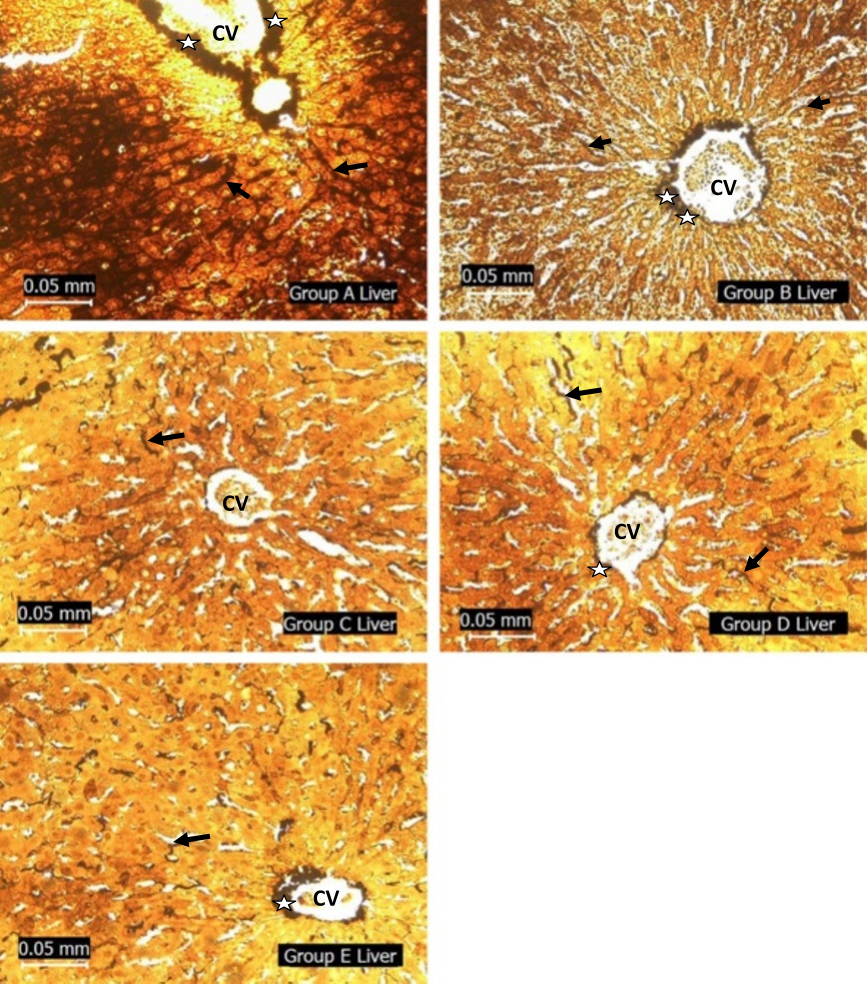
**Photomicrographs showing the liver of the experimental rats (A - normoglycaemic rats, B – test group I, C – diabetic negative control, D – test group II and E – diabetic positive control) stained with Gordon and Sweets reticulin stain.** Note the reticular fibres (stained black) lining the sinusoids (arrow) where they are between plates of liver cells (stained yellow) and also form a dense network (star) around the central vein (CV). The reticular fibres are well stained in the liver section of groups A, B and D rats. However, in groups C and E liver, the reticular fibres are poorly stained except where they form dense network around the central vein.

**Figure 3:**
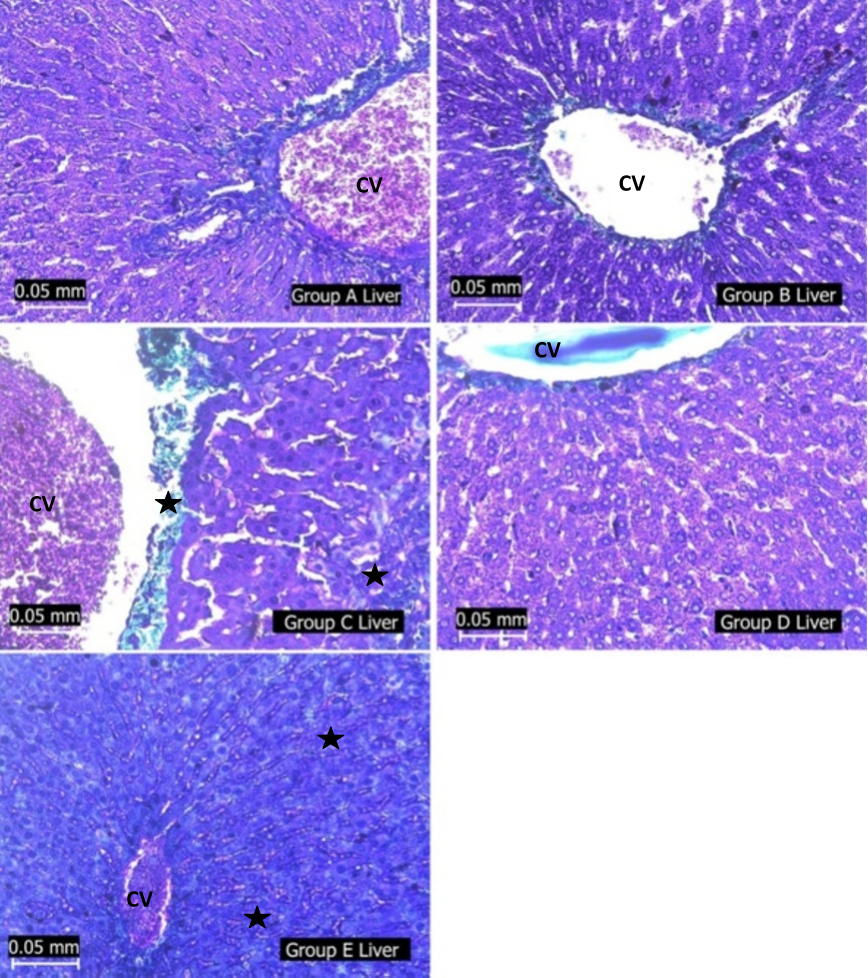
**Photomicrographs showing the liver of the experimental rats (A - normoglycaemic rats, B – test group I, C – diabetic negative control, D – test group II and E – diabetic positive control) stained with Masson trichrome stain.** Note extensive area of collagen staining (black star) around the CV as well as the intercellular matrix around the hepatocytes of the liver groups C and E rats.

**Figure 4:**
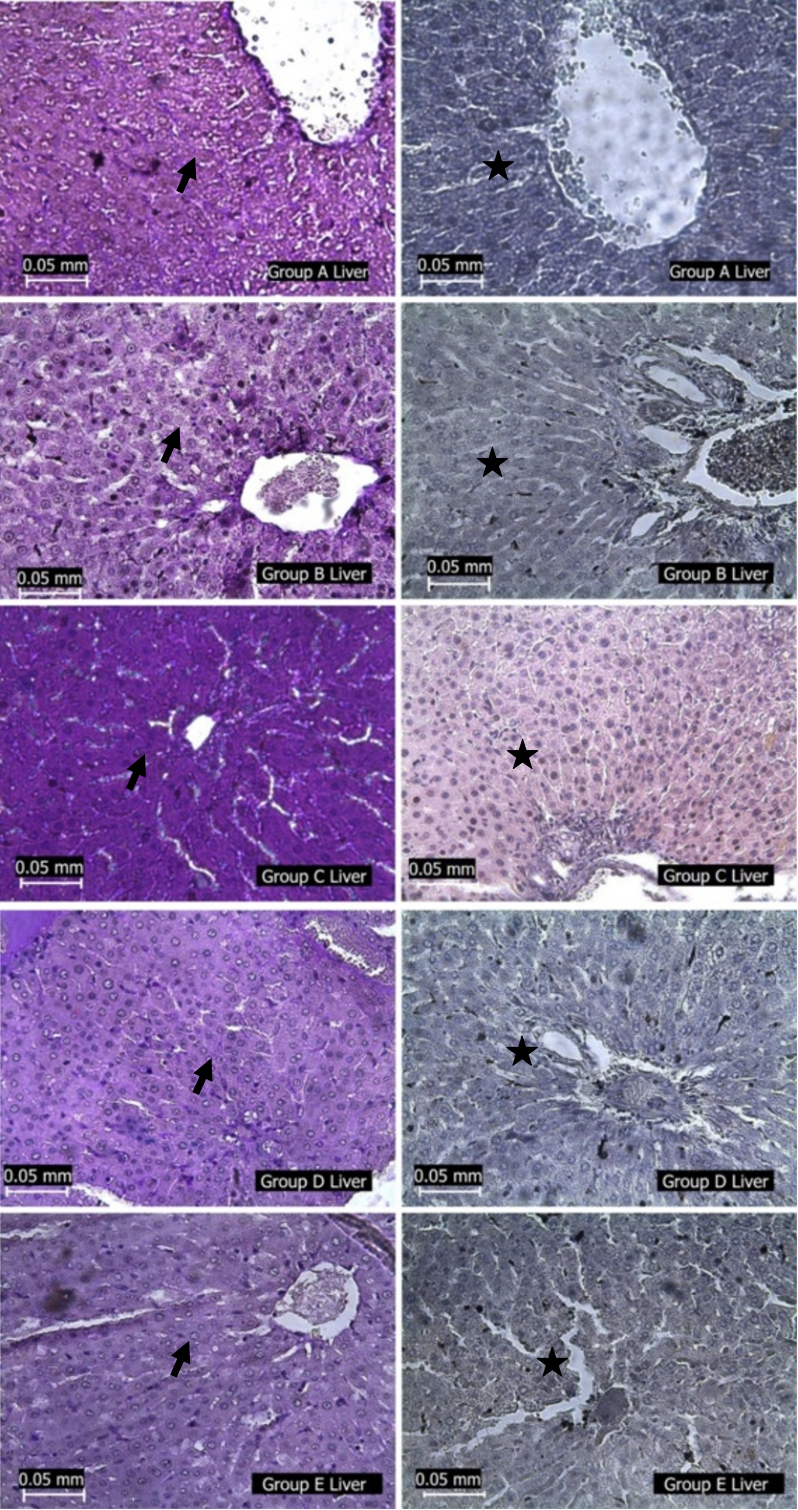
**Photomicrographs showing the liver of the experimental rats (A- normoglycaemic rats, B- test group I, C-diabetic negative control, D test group II, E-diabetic positive control) stained with PAS (arrow) with diastase control (star).** The liver section of group C was deeply stained with PAS while liver sections of other groups were moderately stained with PAS. Diastase negative control was negative to PAS staining in all groups.

Examination of the stained sections of the liver of STZ diabetic rats revealed array of pathological changes including distortion of liver architecture, inflammation as well as necrotic changes (Figure 1), breakdown of reticulin fibres that formed supporting connective tissue of the liver (Figure 2), accumulation of collagen fibres in areas where reticular fibres are broken down (Figure 3) and excessive deposition of glycogen (Figure 4). These pathological changes which persisted in rats treated with protamin zinc insulin were absent in the liver section of diabetic rats treated with HSCE. However, HSCE had no effects on the liver of normal rats.

## Discussion

Diabetes mellitus is associated with progressive metabolic derangement, worsening glycaemic control and morphological changes in the kidney, retina, liver, pancreas and other organs [38, 39]. Oxidative stress is known to play a significant role in the induction of these processes [40]. A number of studies both in vitro and in vivo had shown that extracts of the calyx of *H. sabdariffa* has potent antioxidant activity [41, 42]. This potent antioxidation is thought to form the basis of many of the other healing activities of HSCE including its hepatoprotective activities. Compounds proven to have this antioxidant activity include anthocyanins and protocatechuic acid [43].

The results of this study showed a significant increase in lipid peroxidation in the liver of STZ diabetic rats. This confirms the earlier report on the ability of this diabetogenic compound to induce oxidative damage through generation of free radicals [44]. The increase in lipid peroxidation in the liver may be due to the fact that it contains relatively high concentration of easily peroxidizable fatty acid. Administration of methanolic extract of *H. sabdariffa* reduced MDA, a marker of lipid peroxidation in the liver of diabetic rats suggesting that the extract possesses potent antioxidative properties. The present results are consistent with the report of previous researchers who earlier reported antioxidant effect of extracts from *H. sabdariffa* [41].

In this study, a marked decrease in the activities of catalase (CAT), superoxide dismutase (SOD) and glutathione peroxidase (GPx) in the liver of untreated diabetic rats were observed. The functions of these antioxidant enzymes are interconnected and a decrease of their activities results in the accumulation of lipid peroxides and increases oxidative stress in diabetic rats [45]. Treatment of rats with extracts of *H. sabdariffa* ameliorated the STZ-induced decrease in the concentration of GSH as well as the activities of GPx, SOD and CAT. The results, therefore, lend support to the antioxidant properties of *H. sabdariffa* as demonstrated in both in vitro and various in vivo models [41, 46].

Hepatic cellular degeneration, degeneration of reticular fibres as shown in the silver impregnated liver section (Figure 2) and build up of collagen fibres as shown in the liver sections stained for presence of collagen (Figure 3) revealed the presence of hepatic fibrosis in the liver of STZ-diabetic rats and the insulin treated diabetic rats. The PAS stained sections also revealed an excessive accumulation of glycogen in the liver of the diabetic rats (Figure 4). However, there was restoration of the normal liver morphology in the *H. sabdariffa* – treated diabetic rats. This was shown by a regeneration of reticular fibres as well as reduction in the collagen staining in the liver section of the extract-treated rats. Also, PAS – stained liver section of this group of rats’ reveled glycogen staining that is comparable with that of the normal rats. Histopathological evaluation of the liver of these rats also revealed that *H. sabdariffa* extract also reduced the incidence of liver lesions, including hepatocyte swelling and necrosis induced by STZ.

Hepatic fibrosis is present in various chronic hepatic diseases. It is well known that constant fibrosis can lead to the development of hepatocellular carcinoma [47, 48]. Interrupting and/or reversing hepatic fibrosis may well be a new approach for improving its progression to hepatocellular carcinoma. However, the therapy for reversing liver fibrosis is not yet well established. This study demonstrated that *H. sabdariffa* extract had therapeutic effects on hepatic fibrosis secondary to diabetes induced by STZ in rats. The beneficial effects of *H. sabdariffa* extract in the treatment of chemically-induced fibrosis were evident in liver pathology, as evidenced by decreased severity of the liver morphological changes and fibrosis, generalized improvement of some types of pathological lesions such as fatty liver and cellular degeneration, and reduced hepatic collagen fiber staining. These ameliorative effects of *H. sabdariffa* extract may be mediated by inhibition of hepatic stellate cell activation. In accord with the preventive effects of *H. sabdariffa* extract’s components during the development of chemically induced hepatic damage [20, 21, 43, 49] the present study shows that *H. sabdariffa* extract is effective in the amelioration of STZ-induced liver fibrosis, which would confer a highly significant therapeutic advantage. *Hibiscus sabdariffa* extract is a composite of several antioxidants, such as protocatechuic acid [20, 50] and anthocyanins [21, 22] which prevents peroxidative liver damage.

The activities of AST, ALT and APL increased significantly in diabetic rats in agreement with previous findings [51]. The extract of H sabdariffa significantly reduced the activities of AST and ALT in these rats. This finding is consistent with the finding of Ubani *et al*. [52] who earlier reported that extract of *H. sabdariffa* lowered the activities of these liver marker enzymes in phenobarbitone induced rats.

## Conclusion

In conclusion, this study demonstrated the potentials of *Hibiscus sabdariffa* extract in ameliorating the biochemical and histological changes in the liver of Wistar rats following experimental induction of diabetes mellitus in these rats using STZ. The ameliorative activity of *Hibiscus sabdariffa* extract in the liver of STZ diabetic rats could be partly related to its antioxidant activity and the presence of flavonnoids.

## Abbreviations

HSCE: *Hibiscus sabdariffa* calyx extract
STZ: Streptozotocin
ip: Intraperitoneal
GSH: Glutathione
CAT: Catalase
SOD: Superoxide dismutase
GPx: Glutathione peroxidase
TBARS: Thiobarbituric acid reactive substances
AST: Aspartate amino transferase
ALT: Alanine amino transferase
ALP: Alkaline phosphatase
TP: Total protein
ALB: Albumin
GLB: Globulin
PAS: Periodic acid schiff.
